# The Vagus Nerve in the Neuro-Immune Axis: Implications in the Pathology of the Gastrointestinal Tract

**DOI:** 10.3389/fimmu.2017.01452

**Published:** 2017-11-02

**Authors:** Bruno Bonaz, Valérie Sinniger, Sonia Pellissier

**Affiliations:** ^1^Division of Hepato-Gastroenterology, Grenoble University Hospital, Grenoble, Alpes, France; ^2^U1216, INSERM, GIN, Grenoble Institute of Neurosciences, University Grenoble Alpes, Grenoble, France; ^3^Laboratoire Inter-Universitaire de Psychologie, Personnalité, Cognition et Changement Social LIP/PC2S-EA4145, University Savoie Mont Blanc, Chambéry, France

**Keywords:** vagus nerve, vagus nerve stimulation, cholinergic anti-inflammatory pathway, neuro-immune axis, splenic nerve

## Abstract

The vagus nerve (VN) is the longest nerve of the organism and a major component of the parasympathetic nervous system which constitutes the autonomic nervous system (ANS), with the sympathetic nervous system. There is classically an equilibrium between the sympathetic and parasympathetic nervous systems which is responsible for the maintenance of homeostasis. An imbalance of the ANS is observed in various pathologic conditions. The VN, a mixed nerve with 4/5 afferent and 1/5 efferent fibers, is a key component of the neuro-immune and brain-gut axes through a bidirectional communication between the brain and the gastrointestinal (GI) tract. A dual anti-inflammatory role of the VN is observed using either vagal afferents, targeting the hypothalamic–pituitary–adrenal axis, or vagal efferents, targeting the cholinergic anti-inflammatory pathway. The sympathetic nervous system and the VN act in synergy, through the splenic nerve, to inhibit the release of tumor necrosis factor-alpha (TNFα) by macrophages of the peripheral tissues and the spleen. Because of its anti-inflammatory effect, the VN is a therapeutic target in the treatment of chronic inflammatory disorders where TNFα is a key component. In this review, we will focus on the anti-inflammatory role of the VN in inflammatory bowel diseases (IBD). The anti-inflammatory properties of the VN could be targeted pharmacologically, with enteral nutrition, by VN stimulation (VNS), with complementary medicines or by physical exercise. VNS is one of the alternative treatments for drug resistant epilepsy and depression and one might think that VNS could be used as a non-drug therapy to treat inflammatory disorders of the GI tract, such as IBD, irritable bowel syndrome, and postoperative ileus, which are all characterized by a blunted autonomic balance with a decreased vagal tone.

## Introduction

The vagus nerve (VN), the longest nerve of the organism, makes the link between the central nervous system and the body by innervating major visceral organs such as the heart, the lungs, and the gastrointestinal (GI) tract. The VN is a mixed nerve with 20% efferent and 80% afferent fibers (1), and a major component of the parasympathetic nervous system which composes, with the sympathetic nervous system, the autonomic nervous system (ANS). The sympathetic and parasympathetic nervous systems are classically balanced for maintaining homeostasis. This balance of the ANS is disrupted in various pathologies such as irritable bowel syndrome (IBS), inflammatory bowel diseases (IBD), rheumatoid arthritis (RA), and others, and such an imbalance could also be a predictor of various neuro-immune disorders (2, 3). In particular, an autonomic dysfunction, as represented by a low parasympathetic activity, precedes the development of chronic inflammatory disorders such as RA (4). Consequently, an autonomic dysfunction could be involved in the etiopathogenesis of inflammatory disorders rather than being the consequence of chronic inflammation. The modulation of the ANS, in particular by targeting the VN, is able to improve various pathological conditions such as inflammatory disorders, including IBD, RA, obesity, and pain (5). Such a modulation of the VN is possible through pharmacological manipulation, VN stimulation (VNS), nutritional therapies, physical exercise, and complementary medicines. The VN classically does not innervate lymphoid organs; this role is dedicated to the sympathetic nervous system (6). However, the VN is involved in the neuro-immune axis both through its afferent and efferent fibers. Indeed, the VN stimulates the hypothalamic–pituitary–adrenal (HPA) axis through its afferent fibers to release glucocorticoids by the adrenal glands (7). The VN is also involved in the cholinergic anti-inflammatory pathway (CAP) through a vago-vagal reflex involving a brainstem integrated communication between vagal afferent and efferent fibers i.e., the inflammatory reflex (8, 9). The sympathetic nervous system and the VN interact both through a vago-sympathetic pathway involving vagal afferent fibers (10) and a vago-splenic pathway through vagal efferent fibers (11). Consequently, the VN is at the crossroad of neuro-immune interactions and by stimulating the VN, it is possible to treat various inflammatory disorders of the organism.

In the present manuscript, we will *first*, describe the anatomy of the VN, *second*, characterize the interactions of the VN with the HPA axis and the CAP and the sympathetic nervous system, *third*, explore the interest of therapeutic manipulation of the VN for anti-inflammatory properties through pharmacological activation, VNS, complementary medicines (acupuncture, hypnosis, mindfulness), enteral nutrition, physical exercise, and *fourth*, focus on the role of VNS in the modulation of inflammatory disorder conditions and particularly of the GI tract, such as IBS, IBD, and postoperative ileus (POI).

## Anatomy of the VN

The VN innervates all the GI tract of the rat, except for the rectum (12). In contrast, in human, the GI tract innervation by the VN is debated. For some authors, the VN innervates the digestive tract until the splenic flexure of the colon (13) and the sacral parasympathetic nucleus innervates the rest of the gut through the pelvic nerves; the densest innervation is provided to the stomach. However, the VN could innervate all the digestive tract in human (14). The VN is composed of 80% afferent fibers conveying taste, visceral and somatic information and 20% efferent fibers involved in the control of motility and secretion of the GI as well as cardiac parasympathetic tone (15) and the CAP (8).

Preganglionic neurons of vagal efferents originate in the dorsal motor nucleus of the vagus (DMNV), below the nucleus tractus solitarius (NTS) where vagal afferents project to. A viscerotopic distribution has been described in the rat DMNV such that lateral neurons innervate the stomach while medial neurons innervate the colon (16). Preganglionic neurons are connected with post-ganglionic neurons of the enteric nervous system in the GI tract. Acetylcholine (ACh) is the neuromediator released at both ends of these pre- and post-ganglionic neurons which binds to nicotinic receptors and nicotinic or muscarinic receptors, respectively. The VN is not in direct contact with the intestinal lamina propria (16) but through these enteric neurons (17) which are the effectors of the VN to regulate gut immunity (18).

Vagal afferent fibers originate from the different intestinal layers with their cell bodies located in the nodose ganglia. They end in the NTS according to a rostro-caudal viscerotopic representation (19), and then to the area postrema. The DMNV forms, with the NTS and area postrema, the dorsal vagal complex of the brainstem, a major reflex center of the ANS. Indeed, the activation of vagal afferents generates several coordinated responses (autonomic, endocrine, and behavioral) *via* central pathways involving the dorsal vagal complex. Viscero-sensory informations coming from the NTS to the DMNV influence vagal efferents at the origin of vago-vagal reflexes (20). In addition, the NTS is a relay for these peripheral informations to reach numerous brain areas (21) which compose the central autonomic network (CAN) (22) such as the locus coeruleus (LC), the parabrachial (PB) nucleus the periventricular nucleus of the thalamus, the central nucleus of the amygdala, the paraventricular nucleus of the hypothalamus (PVH), the medial preoptic area, the arcuate nucleus of the hypothalamus, and the ventrolateral medulla (A1-C1 catecholaminergic nuclei) at the origin of an autonomic, behavioral, and endocrine response. The NTS also directly modulates the LC and its projections (23). The rostroventrolateral medulla is one of the two major sources of projections to the LC (24). The latter project to numerous areas of the cortex involved in stress reactions but also in emotional disorders (25). The PVH projects to the bed nucleus of the stria terminalis, the dorsomedial and arcuate hypothalamic nuclei, the medial preoptic area, the periventricular nucleus of the thalamus, the PB region, and the nucleus tegmenti dorsalis lateralis (26). The PB nucleus in return projects to the central nucleus of the amygdala, the bed nucleus of the stria terminalis, and the PVH (27). The PVH projects directly to the NTS (26), thus creating a feedback loop with the forebrain. Consequently, visceral information (e.g., nutrient sensing) driven by the VN is integrated in the CAN involved in the functioning of the ANS and the HPA axis response. The VN is involved in the interoceptive awareness where the insula cortex plays a central role (28). A perturbation of this interoception is observed in diseases of the digestive tract such as IBS but also IBD. Indeed, alexithymia (29) is observed in both of them (30–32).

## The VN and the Neuro-Immune Axis

The VN is a key component of the neuro-immune axis both through its afferent and efferent fibers. The role of vagal afferents was first described by Harris (7) in the regulation of the HPA axis. Indeed, peripheral administration of lipopolysaccharides (LPS), classically used as an experimental model of septic shock, induces the release of interleukin (IL)-1β, a pro-inflammatory cytokine, and finally activates vagal afferents through IL-1 receptors (33). This effect is prevented by vagotomy (34) and works in a dose and receptor-dependent fashion (35). Vagal afferents activate NTS neurons from the A2 noradrenergic group which project to corticotrophin-releasing factor (CRF) neurons of the parvo-cellular PVH. CRF then induces the release of adrenocorticotropic hormone by the pituitary to stimulate the release of glucocorticoids by the adrenal glands to inhibit peripheral inflammation, i.e., the HPA axis.

**Figure 1:**
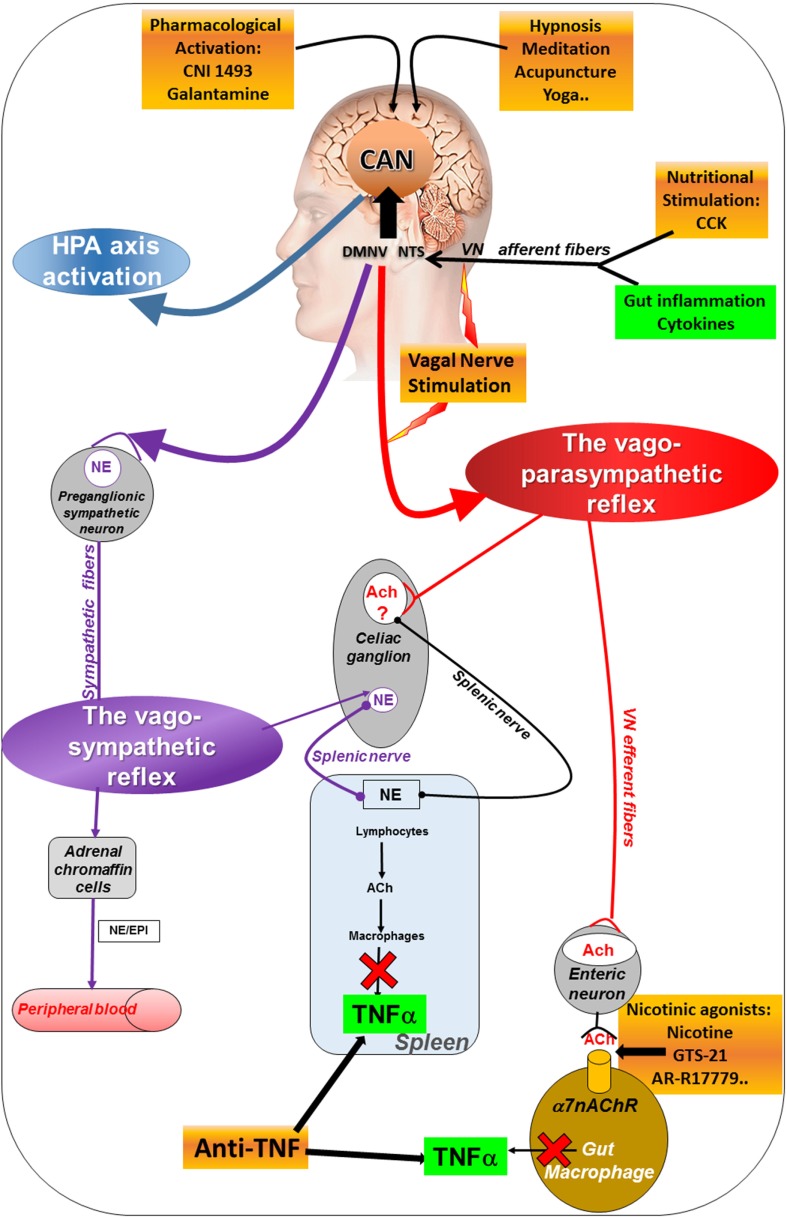
Different pathways of the anti-inflammatory properties of the VN: activation of the HPA axis (blue) through vagal afferents, the cholinergic anti-inflammatory pathway through vago-parasympathetic (red) and sympathetic (purple) reflexes. Targeting the VN for its anti-inflammatory properties (orange) in chronic inflammatory diseases such as inflammatory bowel disease appears as potentially effective therapeutics. Ach, acetylcholine; CAN, central autonomic network; CCK, cholecystokinin; DMNV, dorsal motor nucleus of the vagus nerve; EPI, epinephrine; HPA, hypothalamic–pituitary–adrenal; NE, norepinephrine; NTS, nucleus tractus solitarius; TNFα, tumor necrosis factor-alpha; VN, vagus nerve; α7nAChR, alpha7nicotinic acetylcholine receptor.

In addition to this vagal afferent anti-inflammatory pathway, a second one, described in 2000 by the group of Tracey, involves vagal efferents (36). This group showed that stimulation of the distal end of the VN, i.e., vagal efferents, prevented a LPS-septic shock in rats. VNS had an anti-TNFα effect since liver and blood tumor necrosis factor-alpha (TNFα) levels were dampened. The release of pro-inflammatory cytokines such as TNFα, IL-1β, IL-6, and IL-18 in LPS-stimulated human macrophages was decreased by the release of ACh by the VN. These authors called this pathway “The CAP” (8) (Figure 1) assimilated to an “inflammatory reflex,” i.e., a vago-vagal reflex where the activation of vagal afferents by LPS-stimulated vagal efferents after central integration in the dorsal vagal complex. This group has also identified the α7 nicotinic ACh receptors (α7nAChR) of macrophages involved in this effect (37). de Jonge et al. (38) characterized the cellular mechanistic of this pathway involving α7 subunit-mediated Jak2-STAT3 activation of macrophages and Sun et al. (39) showed that microRNA-124 is responsible of the CAP action by the inhibition of pro-inflammatory cytokines production. The VN is not directly connected with gut resident macrophages but interacts with enteric neurons expressing nNOS, VIP, and ChAT and located within the muscularis next to these macrophages expressing the α7nAChR (40, 41).

The VN has thus a dual anti-inflammatory action both *via* its afferent and efferent fibers activating the HPA axis and the CAP, respectively. Another anti-inflammatory pathway is the vago-splenic pathway.

## The Vago-Splenic Pathway

The group of Tracey also described a vago-splenic pathway, i.e., a vago-sympathetic pathway through the spleen (11). Classically the parasympathetic (i.e., the VN in the present case) and the sympathetic nervous systems have an opposite effect. However, in the vago-splenic pathway, this effect is synergistic through a connection between the VN and the splenic nerve, a sympathetic nerve issued from the celiac ganglion (42), to activate the splenic nerve through the effect of ACh on α7nAChR. The final effect is the inhibition of TNFα release by the spleen (43). A non-neuronal cholinergic pathway is involved in this effect by contrast to the vagal neuronal cholinergic pathway. Indeed, norepinephrine, released by the splenic nerve, binds to β2 receptors of T-lymphocytes of the spleen which release ACh that links to α7nAChR of macrophages to inhibit the release of TNFα by these macrophages (44). These T-lymphocytes are located in the white pulp of the spleen, particularly the central region receiving a dense noradrenergic innervation (45). By comparison to the CAP, there is an intermediate step with a neuro-immune connection involving the splenic nerve and T-lymphocytes. However, the existence of this pathway is still controversial (46) since some authors argue in favor of a direct sympathetic mechanism (47) (see Figure 1). In contrast, the group of Ghia showed that intracerebroventricular injection of a M1 muscarinic ACh receptor agonist activated the CAP; this effect was reversed by vagotomy or splenic neurectomy (48). The same group showed that administration of galantamine, a central ACh-esterase inhibitor activated the CAP and this effect was suppressed by vagotomy, splenic neurectomy, or splenectomy (49). However, a lack of evidence for cholinergic innervation of the rat spleen was reported by Bellinger et al. (50). Martelli et al. (51) argue that the efferent mediator of the CAP is not the VN but the sympathetic nerve, i.e., the splenic nerve. Indeed, they showed that vagotomy has no effect on the LPS-induced TNFα response while both splenic and splanchnic nerves were LPS-activated and suppressed by splanchnicectomy, increasing TNFα levels (46). They evoked a splanchnic anti-inflammatory pathway. In both works of the group of Tracey and Martelli, the model used to activate the CAP and/or the splanchnic pathway was a septic shock induced by LPS which is rather different than other inflammatory conditions in experimental models of IBD and RA. However, both the role of a CAP and a splenic anti-inflammatory pathway are not incompatible when considering a vago-sympathetic pathway involving vagal afferents to the CAN and then descending pathways from the CAN to activate sympathetic nerves.

The sympathetic innervation of the spleen modulates the cellular and humoral immune responses of this lymphoid organ (52–56). Actually, the noradrenergic fibers innervating the spleen (42, 57) are in close contact with immune cells of the white pulp expressing adrenergic receptors (58, 59). The splenic preganglionic neurons located in the thoracic and rostral lumbar spinal cord (60) are controlled by a specific supra-spinal complex circuitry involved in the regulation of neural–immune interactions in the spleen. The VN is able to modulate the sympathetic nervous system after central integration of its afferents in the CAN which is then able to modulate the sympathetic nerves, such as the splenic nerve, through descending pathways from the CAN, i.e., a vago-sympathetic pathway.

## The Vago-Sympathetic Pathway

As described above, vagal afferents end in the NTS and from there activate the CAN which in return is able to modulate the ANS through descending pathways targeting the DMNV and the tractus intermediolateralis in the spinal cord at the origin of vagal and sympathetic efferents respectively. Five brain nuclei of the CAN (i.e., PVH, the A5 noradrenergic group, the caudal raphe region, the rostral ventrolateral medulla, and the ventromedial medulla) modulate the sympathetic outflow (61–63) by innervating preganglionic sympathetic neurons of the intermediolateral cell column in the spinal cord. Hence, the VN could induce a non-direct anti-inflammatory reflex by enhancing the sympathetic outflow. Among these brain structures, the role of the C1 adrenergic group has been recently highlighted by Abe et al. (64) who showed that these adrenergic neurons are involved in the stress protective effect in renal ischemia-reperfusion injury through a sympathetic rather than a vagal pathway. This group had previously shown that activation of vagal afferents or efferents in mice 24 h before injury markedly reduced acute kidney inflammation and TNFα plasma level. This effect was suppressed by splenectomy and was mediated by α7nAChR-positive splenocytes (65). The PVH, through its efferent projections to the DMNV and the spinal sympathetic preganglionic neurons is also able to modulate the ANS. For example, stress through CRF of the PVH, is able to inhibit the DMNV, i.e., vagal efferents, and activate the sympathetic nervous system, i.e., sympathetic efferents (66). Deng et al. (67) have recently shown that chemical stimulation of the hypothalamus protects against colitis in rats through a key role of PVN, NTS and VN. The A5 noradrenergic nucleus of the ventrolateral pons targets almost exclusively the spinal intermediolateral column (68) and is involved in the regulation of visceral sympathetic tone in rodents (69). A5 receives inputs from the C1 neurons (70). The effect of a stimulus on the activity of sympathetic nerves depends on their type fibers composition (71). The relative importance of each of these five regions in the control of the sympathetic outflow may differ. For example, for the spleen, A5 > rostroventrolateral medulla > PVH (71). After pseudorabies injection into the spleen, the A5 region is among the first areas to become infected. Consequently, this region is involved in the response of all sympathetic-innervated organs. A5 neurons must be connected to multiple sympathetic targets. Additional areas may selectively innervate sympathetic preganglionic neurons such as (i) the Barrington’s nucleus exclusively involved in the control of the parasympathetic outflow, (ii) the LC involved in stress and contributing to the generalized sympatho-adrenal activation in response to stressful stimuli, (iii) the periaqueductal gray, lateral hypothalamus, A7 region, NTS, Edinger-Westphal nucleus, pedunculopontine tegmental nucleus, C3 group, caudal ventrolateral medulla, and area postrema (72). Neurons in the rostral ventrolateral medulla increase their activity in association with increases in sympathetic vasomotor reactions (73). All these observations reveal that sympathetic outflow is differentially regulated by supra-spinal areas, without a clearly identified mechanism. Moreover, some areas coordinate global visceral responses (74) thus making it difficult to target specific circuits.

## How to Target the VN for Anti-Inflammatory Properties

The anti-inflammatory properties of the VN could be targeted pharmacologically, with enteral nutrition, by VNS, with complementary medicines or by physical exercise.

### Pharmacological Stimulation of the CAP

Pharmacological stimulation can be obtained by targeting the CAP either centrally or peripherally.

Galantamine, a cholinesterase blocker and a nicotinic receptor agonist, including α7nAChR, is able to cross the blood–brain barrier and activates the central cholinergic pathway thus stimulating VN efferents (75). This drug is used in the treatment of Alzheimer’s disease. Galantamine dramatically decreases circulating TNFα and IL-6 and improves survival in a murine endotoxemia model (75). Thus, galantamine could be used as an immune suppressive drug. To our knowledge, galantamine has only been used in experimental inflammation but not in clinical research. In the same way, CNI-1493 inhibits the p38 MAPK pathway of the TNFα release (76, 77). Central injection of CNI-1493 during endotoxemia significantly reduced serum TNFα levels and this effect is mediated through the VN (9). In a clinical trial, Crohn’s disease (CD) patients who were treated with two doses of CNI-1493 for 2 weeks presented a clinical remission and an endoscopic improvement up to 45% of the patients included (78).

Peripheral α7nAChR can be targeted by agonists such as GTS-21 that was used in a double-blind placebo control trial in experimental human endotoxemia. Healthy volunteers after either GTS-21 or placebo received a low dose of LPS. GTS-21-treated group exhibited lower plasma TNFα, IL-6, and IL-1ra levels compared to placebo (79). In an experimental pancreatitis in mice, pretreatment with GTS-21 significantly decreased pancreatitis severity (80). AR-R17779, another α7nAChR agonist, prevented a mouse model of POI (81).

### Nutritional Stimulation of the CAP

In a model of hemorrhagic shock, enteral nutrition with a high-fat diet induces the release of cholecystokinin (CCK), known to activate CCK1 receptors of vagal afferents, and dampens the inflammatory response (TNFα, IL-6) through a vago-vagal anti-inflammatory reflex (82). In the same study, CCK, vagotomy and nicotinic receptor antagonists prevented the protective effect of high-fat enteral nutrition on intestinal permeability (82). Mucosal mast cells are targets of the nutritional anti-inflammatory vagal reflex since mucosal mast cell degranulation was prevented by lipid-rich enteral feeding (83). Consequently, high-fat enteral nutrition could be used in the treatment of IBD where TNFα and intestinal barrier dysfunction are prominent. Enteral feeding, classically used in the treatment of a flare of IBD, has shown its efficacy to induce clinical remission in CD (84).

### Complementary Medicines

Inflammatory bowel diseases are chronic debilitating diseases with an impact on quality of life and treatments are not always efficient and not devoid of side effects. Consequently, patients often use complementary medicines. Recently, Cramer et al. (85) assessed the efficacy and safety of yoga performed 90 min per week for 12 weeks for improving quality of life in UC patients in clinical remission. By comparison to the written self-care advice group (controls, *n* = 38), the yoga group (*n* = 39) had significantly higher disease-specific quality of life at 12 and 24 weeks of follow-up and disease activity was lower at 24 weeks. Gut-directed hypnotherapy is well known to improve IBS patients (86). Keefer et al. (87) performed seven sessions of gut-directed hypnosis in 26 UC patients in clinical remission vs 29 patients with attention control; the patients were follow-up for 1 year. Patients in the hypnosis group stayed significantly longer in remission at one year than the control group (68 vs 40%). No significant effect has been observed for other psychological factors (quality of life, medication adherence, perceived stress). One mechanism through which complementary medicines may improve IBD could be the activation of the CAP. Acupuncture and meditation reduce both heart rate and inflammatory cytokine release. This effect is mediated by the increase of vagal tone (88). Acupuncture is able to decrease TNFα release following LPS administration in mouse (89). Acupuncture is associated with a down regulation of TNFα synthesis in the spleen that was reversed by splenic neurectomy and vagotomy. Hypnosis modifies heart rate variability (HRV) by enhancing parasympathetic activity and reducing sympathetic tone (90). Yoga (91) and mindfulness meditation (92) increase vagal activity. Consequently, these complementary medicines may be of interest in the treatment of IBD patients *via* the CAP.

### VN Stimulation

In 1880s, Corning JL (93) was the first to use VNS for the treatment of seizures. The technique was then forgotten but reintroduced in 1938 by Bailey and Bremer (94). In 1990, the first VNS for the treatment of pharmacoresistant epilepsy was introduced in human (95) and VNS was approved by the US Food and Drug Administration (FDA) for this indication in 1994 and in 1997 for Europe. In 2005, the FDA approved VNS for the treatment of pharmacoresistant depression (96, 97). Presently, ~100,000 patients have been treated by VNS for epilepsy and ~5,000 for depression (Livanova, Houston, TX, USA).

The antiepileptic and antidepressive effects of VNS can be easily explained by the widespread projections of the VN in the brain from its first relay in the NTS. The mechanism of action of VNS is still not well understood but data argue for a role of the LC, thalamus, hippocampus, periaqueductal gray, and the neocortex (98). If the role of vagal afferent C-fibers was evoked in the antiepileptic effect of VNS, their alteration by capsaicin did not suppress the effect, arguing for a role of vagal A- and B-fibers (99). Five parameters of VNS are classically used: intensity (0.5–3.5 mA), frequency (20–30 Hz), pulse width (250–500 µs), and duty cycle of 30 s ON and 5 min OFF. Frequencies of 2–300 Hz induced electroencephalographic desynchronization of the “encéphale isolé” cat that was dampened by a ligature of the cervical end of the VN (100) thus in favor for a role of vagal afferent fibers. VNS effectiveness is frequency-dependent (101) up to the maximum threshold of 50 Hz beyond which a damage of the VN is induced (102). In rats, VNS (stimulation parameters used for epilepsy) induces neuronal activation in brain area involved in seizures initiation (103). In human, brain imaging studies reported modifications in regions receiving VN afferent supra-medullar projections (104). VNS is a slow-acting therapy since a seizure reduction appears in 50% of patients after 2 years (105). Elliott et al. (106) showed in 65 epileptic patients with a 10-year mean duration of VNS a time-dependent reduction in seizures. Indeed, the positive effect of VNS at 6 months and 1, 2, 4, 6, 8, and 10 years was 35.7, 52.1, 58.3, 60.4, 65.7, 75.5, and 75.5%, respectively.

Vagus nerve stimulation can be applied invasively or non-invasively through the skin. Invasive VNS is classically performed under general anesthesia by a neurosurgeon and an electrode is wrapped around the left cervical VN in the neck connected subcutaneously by a cable to a pulse generator located in the left chest wall (107). The implantation lasts ~1 h. VNS is classically performed onto the left VN which innervates the atrioventricular node of the heart while the right VN innervates the sinoatrial node thus with a weaker influence on the heart rate (108). The VNS device is manufactured by Livanova, a merger of Cyberonics and Sorin (Houston, TX, USA), and composed of a pair of helical electrodes (2 or 3 mm diameter), a battery-powered generator, a tunneling tool, software and programming tools (www.livanova.com/). The price of the generator pulse (model 102) plus the electrode (model 302) is ~9,300 €. Safety and tolerability were demonstrated for implantable VNS (101). The minor adverse events which are classically reported by the patients are: voice alteration, cough, dyspnea, paresthesia, nausea, headache and pain; these adverse events decline over time and are easily controlled by reducing stimulation intensity (109). The battery life depends on the frequency of stimulation used and is longer for low frequency (5–10 Hz), e.g., ~5–10 years, than high frequency (20–30 Hz).

Based on the concept that the CAP involves parasympathetic outflow of the vagal nerve, VNS is performed at the lowest frequencies (1–5–10 Hz) to produce its anti-inflammatory effect. Borovikova et al. (36) performed low frequency (1 Hz) VNS in rats with cervical vagotomy and stimulated the distal end cut of the VN thus stimulating vagal efferents. Bernik et al. (110), who performed VNS of the left or right VN in anesthetized rats, demonstrated that a 20 min-stimulation prevented endotoxin-induced hypotension.

Non-invasive VNS (n-VNS) does not need surgical implantation and improves the safety and tolerability of VNS. Transcutaneous auricular VNS (ta-VNS) is one of these techniques. Indeed, the VN includes a sensory “auricular” branch that innervates exclusively the cymba concha of the external ear (111) and projects to the NTS in cats (112) and humans (113). ta-VNS produces the same cognitive and behavioral effects than VNS (114). When performed at 25 Hz in healthy adults, it affects the vagal central projections, compared to a control stimulation in the earlobe (113). The close anatomical connection between auricular concha, VN, NTS, and DMNV can thus explain the auricular-vagal reflex. Consequently, ta-VNS could activate the anti-inflammatory pathway. In agreement with this neuroanatomical concept, ta-VNS suppresses LPS-induced inflammatory responses *via* α7nAChR in rats (115) and this effect was suppressed after vagotomy or with α7nAChR antagonist injection.

Presently, there are two n-VNS devices that are used for epilepsy, depression, and headache but which could also be used in inflammatory disorders of the GI tract such as IBD, IBS, and POI as well as others. The Cerbomed device called NEMOS (Erlangen, Germany) uses an intra-auricular electrode (like an earpiece) to stimulate the vagal auricular branch (116) and has received the European clearance in 2011 for the indication epilepsy. This device is available in Austria, Germany, Italy, Switzerland, and UK. The optimal stimulation is chosen by the patients based on the intensity to feel a non-painful stinging with a recommended stimulation duration of 4 h per day. A 70% reduction seizure frequency was observed after 9 months of ta-VNS (116) and a 43% reduction has been observed after 8 weeks in another study (117). ta-VNS was shown to increase HRV and reduce sympathetic outflow in controls (118). The second device is referred as GammaCore (electroCore LLC, Basking Ridge, NJ, USA) and comprises a portable stimulator and two stainless steel round disks functioning as skin contact surfaces that deliver a locked, low-voltage electrical signal to the cervical vagal nerve; each stimulation cycle lasts 120 s. An improvement of headache was reported in 48% of patients (119). In another study, mean pain scores were significantly reduced at 2 h from baseline in patients with chronic migraine (120). GammaCore is presently evaluated in controlled trials in North America and EU in patients with primary headache disorders. n-VNS with the Gammacore system decreases whole blood culture-derived cytokines and chemokines in healthy volunteers (121). No significant serious device-related adverse events have been reported with NEMOS and Gammacore. By comparison to invasive VNS, n-VNS has the disadvantage of its compliance which is an important problem in the treatment of chronic inflammatory diseases.

### Physical Exercise

An imbalance of the ANS, with low vagal and high sympathetic activities, correlates with numerous pathological conditions such as arrhythmia, heart failure, and hypertension and ischemia/reperfusion injury. Cardiovascular morbidity and mortality and inflammation are all decreased by high levels of cardiorespiratory trainings (122, 123). There is a negative correlation between cardiorespiratory fitness and cardiovascular events, partly mediated by inflammatory factors (124). The ANS is known to affect the relation between cardiorespiratory fitness and inflammation in middle-aged men. Then, physical activity and exercise training may exert a stimulatory effect on the CAP since RR variability is inversely related to inflammatory markers (125). Regular physical exercise induces an increase in resting vagal tone (126) and increases central 5-HT synthesis and central 5-HT increases vagal modulation in conscious rats (127).

## VN in the Modulation of Inflammatory Disorder Conditions

Based on its activation of the HPA axis and the CAP, the VN has the ability to modulate inflammatory conditions. Experimental and more recently clinical data involving pilot studies are available for this effect in the domain of IBD, RA, and POI. In the next lines, we will focus on GI inflammatory disorders such as IBD, IBS, and POI.

### Chronic Inflammatory Bowel Disorders

Inflammatory bowel diseases are classically represented by CD and ulcerative colitis (UC). CD involves all the digestive tract and ano-perineal region while UC involves the recto-colon. IBD begin between 15 and 30 years and are characterized by alternation of flares and remissions. During flares, patients have several intestinal and extra-intestinal symptoms such as abdominal pain, diarrhea, skin, eyes, or joints inflammation thus explaining their significant impact on the quality of life of IBD patients. Both CD and UC are heterogeneous in their natural history (128). About 1.5 million Americans and 2.2 million Europeans are affected by IBD (129) and there is an increase of the incidence and prevalence of IBD due to the “Westernization” of our lifestyle. Immunologic, genetic, and environmental factors are involved in the pathophysiology of IBD (130). Experimental and clinical data seem to show a role of stress in the pathophysiology of IBD (131). Classically, stress increases intestinal permeability, modify intestinal microbiota and immunity which are factors involved in the pathophysiology of IBD. The VN is involved in the stress effects on the digestive tract. Indeed, stress classically inhibits the VN and stimulates the sympathetic nervous system (66). Chronic stress instead of acute stress is more involved in the pathophysiology of IBD as well as others GI disorders such as IBS (132). Stress induces an imbalanced ANS as reported in IBD with a blunted sympathetic activity in CD (133) and a vagal dysfunction in UC (134). We previously reported a relationship, in IBD patients, between an imbalanced ANS, psychological adjustment (3) and pro-inflammatory profiles (135). Presently, standard treatment of IBD patients is represented by steroids, immunosuppressants (thiopurines, methotrexate), biologicals (anti-TNFα, anti-adhesion molecule, anti-IL12/23). The therapeutic goal is not just to relieve IBD-related symptoms but also to favor mucosal healing because it has been involved in a superior long-term prognosis including a lower surgical risk, hospitalizations, and need for systemic steroids (136). Anti-TNFα therapies have changed the prognostic of IBD but 10%-40% of patients lose response within 12 months (137) and a further 10–20% annually thereafter (138). In addition, these treatments are not devoid of side effects (139) and adherence to medications is a challenge in IBD patients (140). Surgery for IBD occurs for 70% of CD patients and 35% of UC patients (141). Surgical operation is performed in case of failure of medical treatment or complications and patients are re-operated because surgery, but also medical treatment, is not curative but only suspensive. The diagnosis of IBD is often done late at a time where lesions are evolved such as stenosis, fistula, abscesses in CD, and more refractory to medical treatment. Consequently, targeting IBD early when the disease is purely inflammatory is of interest. These patients have also a risk of recto-colonic cancer due to chronic inflammation and mucosal healing is presently a gold standard in the treatment of IBD.

#### Experimental

The VN anti-inflammatory activity potentiating the CAP has been reported in experimental colitis (142, 143), after vagotomy (142), VNS (144, 145), and peripheral or central injection of AChesterase inhibitors (146). Its anti-inflammatory role goes through a macrophage-dependent mechanism involving nicotinic receptors. However, other counter-inflammatory mechanisms play also a role when vagal integrity is compromised and does not play its protective role (147).

Classically, low frequency (5–10 Hz) VNS is known to stimulate vagal efferents, i.e., the CAP. However, we have shown in experimental conditions that even at low frequency stimulation vagal afferents are also activated in anesthetized rats under VNS in an fMRI study using dynamic causal modeling to estimate neuronal connectivity (148). We have also reported that long-term low frequency (10 Hz) VNS was able to induce modifications of the electroencephalogram in a CD patient under VNS (149). In fact, in the neuroanatomic context of the pathways that are involved in the anti-inflammatory role of the VN both stimulation of vagal afferent and efferent fibers is of interest.

Using VNS in a rat model of TNBS colitis classically used for CD, we have shown that low frequency (5 Hz) chronic VNS performed for five consecutive days with parameters classically used for epilepsy improved colitis (144). Indeed, a multiparametric index of colitis taking into account clinical, biological, macroscopic and histological damage, as well as pro-inflammatory cytokines, was improved in rats under VNS. We observed that VNS was more efficient on the area of lesion with less inflammation located immediately above the principal inflammatory lesion. In the same experimental colitis model, Sun et al. (145), have also evaluated the chronic VNS effect but with a higher frequency stimulation (20 Hz) on colonic inflammation using clinical, histological, and biochemical parameters. They also recorded HRV in rats with colitis under VNS. They observed a significant decrease of colitis under VNS and IL-6 and TNF-α cytokines, and show an improvement of the sympatho-vagal balance. Very recently, in a similar approach, Jin et al (150), using the same model of TNBS colitis, showed that chronic VNS improved colonic inflammation by inhibiting pro-inflammatory cytokines *via* the autonomic mechanism; addition of non-invasive electroacupuncture to VNS enhanced the anti-inflammatory effect of VNS.

#### Clinical

Until recently, only few data were available concerning the anti-inflammatory role of the VN in IBD. However, recording of vagal tone and the sympatho-vagal balance using HRV, a reliable non-invasive tool that quantifies sympathetic and parasympathetic activities, allows such an approach. The risk for developing a chronic disease is associated to a dysregulated ANS with a decreased vagal tone. In the context of brain-viscera interaction, HRV monitoring is an important tool which allows the sensing of vagal tone and its impairment and, hence, the CAP deficiency. HRV monitoring is a biomarker which predicts the prognosis of several chronic inflammatory diseases (151). As we know that a decrease in vagal tone induces a reduction in HRV. We have shown in IBD patients a correlation between vagal tone and emotional adjustment (low negative emotions vs high negative emotions) and the way of how patients coped with their disease. A positive coping profile was associated with a low vagal tone in CD and with a high vagal tone in UC (3). Consequently, it is important to separate IBD patients according to the disease (CD vs UC) as well as the importance of psychological factors on vagal tone. In addition, recent data have shown that an autonomic dysfunction precedes the development of RA (4). We have also reported that CD patients with a low resting vagal tone presented higher blood TNFα and salivary cortisol levels than patients with high vagal tone (135). A low vagal tone is thus associated with a pro-inflammatory state. In addition, based on the fact that stress inhibits the VN and thus favors a pro-inflammatory state, this may explain, at least in part, that stress could favor a relapse in IBD patients. In this context, monitoring resting vagal tone over time could be useful (a) for predicting vulnerable state, (b) for proposing adapted enforcement therapy such as complementary medicine, known to stimulate the VN, pharmacological manipulation of the CAP, or VNS to restore a normal vagal tone, and (c) for a follow-up of the therapy efficacy on the parasympathetic system.

In a translational approach in CD patients, we have performed a pilot VNS study where 7 patients with active ileo-colonic CD where implanted with a VNS device. Only two patients out of seven were on treatment (Azathioprine) on inclusion. We have recorded clinical (Crohn’s disease activity index, CDAI), biological (CRP, fecal calprotectin), endoscopic (Crohn’s disease endoscopic index of severity, CDEIS) markers of activity during a 6 months of follow-up. The first implanted patient was on April 2012 and the 7th patient on November 2014. All the patients entered in a follow-up study. VNS induced deep remission in five of the seven patients. Two patients were taken off the study after a 3 months VNS and switched to infliximab and azathioprine, one was operated (ileo-cecal resection). These two patients had the highest CDAI, CRP and CDEIS on inclusion which suggests that VNS, as a slow-acting therapy, is more indicated in moderate CD. All the patients have kept the device in place with the duty cycle still running, except one of the two patients removed from the study who have a low intensity of stimulation (0.5 mA). VNS was well tolerated with the classical minor side effect represented essentially by hoarseness. We did not have any problem of infection either local or systemic and no VNS device was removed. The data on the first seven patients after a 6-month follow-up were reported for the first time recently (152). VNS could also be used to maintain remission induced by drugs. Surgery is used to cure CD lesions and VNS as a slow-acting therapy could be an interesting tool to prevent postoperative recurrence of CD.

### Irritable Bowel Syndrome

Abdominal pain, bloating and altered bowel habits without any organic cause with a higher prevalence in women (153) are the main characteristics of IBS. IBS prevalence goes from 10 to 15% in industrialized countries (154) and represents up to 12% primary care doctors and 28% gastroenterologist medical visits (155). Significant impairment in quality of life, time off work, and significant increase in health care costs are the principal consequences of IBS. Extra-intestinal manifestations such as headache, arthralgia, urinary problems, insomnia, and fatigue are classically reported by the patients in association with digestive symptoms. Fibromyalgia, frequently associated with IBS, worsens digestive symptoms (156). Psychological factors as anxiety or major depression, are often observed in IBS patients (up to 50%) (157). Stress has a major role in the pathophysiology of IBS (132). In particular, early life trauma such as a history of emotional, sexual, or physical abuse is reported in 30–50% of patients (158) and symptoms are often triggered by stress. Intestinal distension–induced visceral hypersensitivity and characterized by lower pain thresholds is often observed in IBS patients and is a classical marker of the disease (159). Mechanisms of this visceral hypersensitivity seem to be explained by a low-grade inflammation in the GI tract (that could favor modifications of neuronal plasticity) (160) and by a mast cells sensitization of intestinal afferent terminals (161). Bacterial gastroenteritis is associated with 4–30% of post-infectious IBS (162). However, anxiety, high levels of perceived stress, somatization and negative illness beliefs at the time of infection were also predictors of post-infectious IBS (163), arguing for a cognitive-behavioral model of IBS. IBS has been compared to an IBD “a minima” since an increased number of gut mucosal T-lymphocytes and mast cells as well as an increased of blood level pro-inflammatory cytokines (IL-10 and IL-12, suggesting Th1 polarization) have been described (164). Globally, IBS is described as a biopsychosocial model due to a blunted brain–gut axis consistent with an up-regulation in neural processing between gut and brain. Patients are hypervigilant toward their symptoms explaining visceral hypersensitivity. Central sensory processes are modified in IBS patients (165) and this is assimilated to a central sensitization syndrome (166, 167). Dysautonomia, a marker of brain-gut dysfunction, has been described with a high sympathetic and a low parasympathetic tone, irrespective to the positive or negative affective adjustment (3). Because of the multifactorial pathophysiology of IBS, its medical treatment is disappointing and essentially based to alleviate symptoms. Psychotherapy, like cognitive-behavioral therapy and complementary medicine like hypnosis, are known to improve vagal tone (90, 168, 169), and could be of interest in the treatment of IBS symptoms.

From a pathophysiological point of view, targeting both the GI tract and the central nervous system through the VN is of interest in IBS. Based on its peripheral anti-inflammatory action through the CAP and on its central effect, as antidepressive, VNS would be of major interest in IBS treatment. In addition, the VN is involved in the control of pain and VNS has been shown to modify central pain processing. Indeed, in visceral pain models in rats, VNS has been shown to increase the pain threshold (170) and to modulate visceral pain-related affective memory (171). Modification of pain by VNS has also been reported in epileptic patients certainly by modulating peripheral nociceptor function (172). Deep breathing increases cardiac vagal tone and prevents the development of acid-induced esophageal hypersensitivity in healthy volunteers; this effect was abolished by atropine (173). Somatic pain thresholds are increased in healthy volunteers with ta-VNS (174). VNS activates vagal afferents that project to brain nuclei involved in the descending inhibitory modulation of pain (175).

Presently, there is no published data on the treatment of IBS by VNS although two studies using n-VNS are registered in ClinicalTrial.gov. The first study has been set up by ElectroCore LLC, with a new n-device called GammaCore. This randomized, single center, double-blind, parallel, sham-controlled pilot study relates on the treatment of symptoms caused by functional dyspepsia or IBS (ClinicalTrials.gov Identifier: NCT02388269). Although completed, no results have been still posted. The second study, still recruiting, evaluates the effect of a 6-month transcutaneous VNS on intestinal and systemic inflammation, intestinal transit time mucosal permeability, and quality of life in IBS patients (ClinicalTrials.gov Identifier: NCT02420158). Ten IBS women, aged between 18 and 60 years, will be included.

### Postoperative Ileus

Abdominal surgery induces POI whatever the localization of surgery site. POI is defined by a delayed gastric emptying and a prolonged intestinal transit (176). Stomach and small intestine functions turn back to normality within 24–48 h while the colon takes generally more time (up to 72 h). The recovery of GI motility can take longer hospitalization times and thus higher healthcare costs. The cost of this postoperative complication has been estimated at US$1 billion/year in the US (176). Sympatho-adrenergic and vagal nonadrenergic noncholinergic inhibitory efferent pathways play a role in the POI mechanisms while capsaicin-sensitive neurons are implicated in the afferent pathway of the reflex (177). Supra-spinal brain nuclei have also been implicated in POI, in particular, specific hypothalamic and pontine-medullary neurons involved in the autonomic regulation of GI function (178). A role for CRF in the PVH is evoked since CRF is a key mediator in the stress effect on the GI tract. Indeed, stress is well known to inhibit gastric emptying (179) as shown by the intracerebroventricular injection of a-helical CRF-(9–41), a CRF antagonist, which reduces the delay of gastric emptying under stress conditions (180). This effect is CRF1 receptor-dependent. More recently, a peripheral pathway, involving the CAP, has been described in the mechanism of POI. Indeed, abdominal surgery induces inflammation of the muscularis propria (181) and activation of resident macrophages which release TNFα. Depletion and inactivation of the muscularis macrophage network prevents POI. Systemic administration of selective nACh agonists as well as VNS reduces the inflammatory response to manipulation of the intestine during surgery (81). This anti-inflammatory effect, mediated by a reduction in macrophage activation and cytokine production is driven by the CAP (48). Gum chewing reduces POI by stimulating vagal activity (182). Targeting the CAP could thus improve POI by its anti-inflammatory action and VNS could therefore be a potential treatment to prevent POI.

In a mouse model of intestinal manipulation, de Jonge et al. (38) have shown that 5 min of cervical VNS prior to abdominal surgery improved GI transit through alpha7 subunit-mediated Jak2-STAT3 activation in intestinal macrophages, indicating that VNS may represent a new therapeutic approach to shorten POI. Stakenborg et al. (183) have recently explored the therapeutic potential of VNS in patients undergoing abdominal surgery for colo-rectal cancer, randomized to sham stimulation (*n* = 5), 5 Hz stimulation (*n* = 6), or 20 Hz stimulation (*n* = 7) group. They performed 1 ms and 2.5 mA during 2 min of VNS at the beginning and at the end of the surgery. They showed that abdominal VNS significantly reduced LPS-induced IL8 and IL6 production by whole blood in patients. In the same study, they showed that abdominal VNS was as potent as that of cervical VNS in a murine model of POI.

## Conclusion

Through the HPA axis and the CAP, the VN exerts an anti-inflammatory action. There is also an anti-inflammatory vago-sympathetic pathway where the VN and the sympathetic system (i.e., the splenic nerve) act synergistically. This anti-inflammatory effect involves both vagal afferent and efferent fibers. Targeting the VN opens new therapeutic avenues in GI inflammatory diseases such as IBD, POI, IBS, and other TNFα-mediated diseases such as RA or psoriasis. Among these therapeutic approaches, VNS, either invasive or non-invasive, appears as an interesting tool with no major side effects in the era of Bioelectronic Medicine (184). Patients with chronic diseases are open to such a non-drug therapy because they are more and more reluctant to conventional therapies in particular because of their side effects and the need of chronic use of these treatments.

## Author Contributions

BB wrote the first draft of the manuscript and VS and SP provided critical feedback to improve it.

## Conflict of Interest Statement

The authors declare that the research was conducted in the absence of any commercial or financial relationships that could be construed as a potential conflict of interest.
